# From Synaptic Dysfunction to Neuroprotective Strategies in Genetic Parkinson’s Disease: Lessons From LRRK2

**DOI:** 10.3389/fncel.2020.00158

**Published:** 2020-07-28

**Authors:** Andrea Mancini, Petra Mazzocchetti, Miriam Sciaccaluga, Alfredo Megaro, Laura Bellingacci, Dayne A. Beccano-Kelly, Massimiliano Di Filippo, Alessandro Tozzi, Paolo Calabresi

**Affiliations:** ^1^Section of Neurology, Department of Medicine, University of Perugia, Perugia, Italy; ^2^Section of Physiology and Biochemistry, Department of Experimental Medicine, University of Perugia, Perugia, Italy; ^3^Oxford Parkinson’s Disease Centre, Department of Physiology, Anatomy and Genetics, University of Oxford, Oxford, United Kingdom; ^4^Neurologia, Fondazione Policlinico Universitario Agostino Gemelli IRCCS, Rome, Italy; ^5^Neuroscience Department, Università Cattolica del Sacro Cuore, Rome, Italy

**Keywords:** Parkinson’s disease, LRRK2, synaptic dysfunction, mitochondrial dysfunction, α-synuclein, neuroprotection

## Abstract

The pathogenesis of Parkinson’s disease (PD) is thought to rely on a complex interaction between the patient’s genetic background and a variety of largely unknown environmental factors. In this scenario, the investigation of the genetic bases underlying familial PD could unveil key molecular pathways to be targeted by new disease-modifying therapies, still currently unavailable. Mutations in the leucine-rich repeat kinase 2 (LRRK2) gene are responsible for the majority of inherited familial PD cases and can also be found in sporadic PD, but the pathophysiological functions of LRRK2 have not yet been fully elucidated. Here, we will review the evidence obtained in transgenic LRRK2 experimental models, characterized by altered striatal synaptic transmission, mitochondrial dysfunction, and α-synuclein aggregation. Interestingly, the processes triggered by mutant LRRK2 might represent early pathological phenomena in the pathogenesis of PD, anticipating the typical neurodegenerative features characterizing the late phases of the disease. A comprehensive view of LRRK2 neuronal pathophysiology will support the possible clinical application of pharmacological compounds targeting this protein, with potential therapeutic implications for patients suffering from both familial and sporadic PD.

## Introduction

Parkinson’s disease (PD) represents one of the most common neurodegenerative disorders of the central nervous system (CNS; Dorsey et al., 2007; Kalia and Lang, 2015; Ascherio and Schwarzschild, 2016). The prevalence of PD has been reported to be higher in Europe and Northern America, with respect to African, Asian, and Arabic countries (Kalia and Lang, 2015). Overall, PD is thought to affect a number of people ranging from 66 to 1,500 per 100,000 in Europe (von Campenhausen et al., 2005) and from 111 to 329 per 100,000 in Northern America (Strickland and Bertoni, 2004). The incidence of PD is strictly dependent on demographic factors, with an exponential increase after 80 years of age (Driver et al., 2009), a male-to-female ratio of 3:2 (de Lau and Breteler, 2006), and a higher number of cases among Hispanics and non-Hispanics white Americans (Van Den Eeden et al., 2003). Taking into account the expected progressive population aging, the number of patients suffering from PD is thought to significantly increase in the next decades, making this disease one of the main health issues to be faced in the future. Unfortunately, despite the availability of various symptomatic treatments (Connolly and Lang, 2014), effective disease-modifying therapies aimed at blocking or slowing down the progression of the disease are still lacking.

In this scenario, the development of new effective therapeutic strategies requires a better understanding of the pathogenetic processes leading to PD. The main histopathological features of PD are represented by the progressive loss of dopaminergic (DAergic) neurons in the midbrain substantia nigra pars compacta (SNpc) and the accumulation of intraneuronal insoluble protein aggregates named Lewy bodies (LBs; Kalia and Lang, 2015; Poewe et al., 2017). Since their discovery, the pathologic pathways leading to the formation of LBs have been considered crucial processes to be decrypted in order to unveil the pathogenesis of PD. The effort dedicated to the investigation of the molecular composition of LBs led to the identification of an abnormally folded protein as their main component, α-synuclein (α-syn; Goedert et al., 2013). The physiological functions of α-syn, which are still under investigation, include a wide range of neuronal homeostatic processes, such as synaptic vesicle dynamics and mitochondrial activity regulation (Vekrellis et al., 2011; Wales et al., 2013; Burré, 2015).

The basal ganglia network was traditionally depicted as divided in two structurally and functionally separated pathways, one favoring (direct) and one inhibiting (indirect) locomotor activation and movements (Calabresi et al., 2014). The projections arising from the SNpc can modulate the activation of the direct and indirect pathway and, specifically, dopamine (DA) can favor the movement through the activation of DA D_1_ receptor (D_1_R) mainly expressed by striatal spiny projections neurons (SPNs) of the direct pathway. Conversely, SPNs of the indirect pathway preferentially express DA D_2_ receptor (D_2_R), which exerts a neuronal inhibitory effect (Calabresi et al., 2014). It should be noted that recent findings have suggested a more complex and less simplistic view of the basal ganglia network, in which the DAergic regulation of striatal synaptic plastic properties is crucial to maintain a physiological motor function (Calabresi et al., 2014). Based on this, the loss of the regulatory role played by the DAergic nigral projections to the nucleus striatum causes a dysfunction of the whole basal ganglia neural circuit, paralleled by the occurrence of the typical parkinsonian motor syndrome.

The characteristic PD clinical picture was described by James Parkinson almost 200 years ago and includes bradykinesia, muscular rigidity, resting tremor, and postural and gait impairment (Przedborski, 2017). Over time, it became clear that PD patients are also characterized by a multitude of nonmotor features, which can precede the onset of motor symptoms by several years, such as depression, hyposmia, constipation, and sleep disorders (Kalia and Lang, 2015; Schapira et al., 2017). In this context, it should be noted that, among PD-related nonmotor features, the presence of cognitive impairment and autonomic dysfunction could have a dramatic impact on patients’ quality of life (Poewe et al., 2017; Schapira et al., 2017). These observations have radically changed the pathogenic view of PD, suggesting an involvement of different brain areas at different times during the course of the disease, even before the loss of nigral neurons. While as previously mentioned the classical avenue of research has focused on the hallmark degeneration of the SNpc, the identification of early nonmotor features suggests a functional impairment of different brain areas at different times during the course of the disease, anticipating degeneration by several decades. Indeed, aberrant neuronal specific functions such as synaptic efficacy and neurotransmission represent an early temporal window that can be exploited for therapeutics benefit. In addition, the loss of DAergic cells was reported not to be complete at the onset of the parkinsonian motor syndrome (Kordower et al., 2013), also suggesting the presence of a therapeutic window in which the administration of neuroprotective drugs could significantly ameliorate the prognosis of patients suffering from the premotor and early motor PD phases. In this scenario, the identification of the mechanisms triggering PD-related neurodegenerative process is mandatory.

After almost two centuries of research in this field, PD is considered a multifactorial disease in which genetic and environmental factors synergistically trigger the disruption of multiple cellular processes, such as mitochondrial activity, synaptic transmission, and protein degradation pathways (Kalia and Lang, 2015). Several environmental toxins, herbicides, or pesticides have initially catalyzed the attention of the scientific community as possible etiologic factors, leading to the development of multiple toxin-based experimental models of PD (Goldman, 2014). However, the intense investigation of the genetic abnormalities underlying PD development has changed the etiologic view of the disease.

Genome-wide complex trait analysis suggested that the potential heritable factors leading to PD account for 30% of the total risk, with 28 identified genetic loci and many still unknown abnormalities that remain to be discovered/identified (Keller et al., 2012; Wood et al., 2013). The genetic abnormalities associated with a high risk of disease development, underlying almost 5% to 10% of all PD cases, are often called monogenic or causative mutations (Keller et al., 2012). By now, the list of genes involved in PD pathogenesis includes genes responsible for autosomal dominant PD (such as PARK1/SNCA, LRRK2, VPS35, EIF4G1, DNAJC13, and CHCHD2), autosomal recessive PD (such as Parkin, PINK1 and DJ-1) or less typical parkinsonian syndromes (for instance, PLA2G6 or ATP13A2; Kalia and Lang, 2015). Among these, PARK1/SNCA gene encoding α-syn was the first to be identified as responsible for autosomal dominant PD in Italian and Greek families (Polymeropoulos et al., 1997; Verstraeten et al., 2015). Point missense mutations (including the A30P, A53T and E46K), duplication, or triplication of this gene confer high risk of PD development (Singleton et al., 2003; Vekrellis et al., 2011; Kara et al., 2014; Verstraeten et al., 2015), reinforcing the idea that α-syn aggregation plays a crucial role in the pathogenesis of the disease (Surmeier et al., 2017).

A particular interest has grown around the gene encoding leucine-rich repeat kinase (LRRK2), localized in the PARK8 locus, because its mutations can account for approximately 4% of all familial PD cases, representing the most frequent genetic cause of PD, and can be also identified in approximately 1% of sporadic cases (Healy et al., 2008). LRRK2 is a large protein, weighing 280 kDa with more than 2,500 amino acids, characterized by different domains such as leucine-rich repeats, WD40, Ras of complex-carboxy terminal of Roc (Roc-COR) GTPase, and serine–threonine kinase domains (Mata et al., 2006; Cookson, 2010). LRRK2 is expressed throughout the body and the brain, in many cell types; it is enriched in axonal and dendritic processes of cortical and striatal neurons, with a lower expression in DAergic nigral cell bodies (Melrose et al., 2006, 2007; Lee et al., 2010).

The functions of this protein have been extensively investigated, suggesting its involvement in a wide range of physiological processes including synaptogenesis and immune system modulation (Saha et al., 2009; Cookson, 2010, 2012; Piccoli et al., 2011; Dzamko and Halliday, 2012; Lee S. et al., 2012; Sanna et al., 2012; Russo et al., 2014; Taymans et al., 2015; Wallings et al., 2015; Roosen and Cookson, 2016; Rassu et al., 2017; Price et al., 2018). A temporal increase of LRRK2 levels in both primary culture and tissues (Biskup et al., 2006; Piccoli et al., 2011; Beccano-Kelly et al., 2014) illustrates a probable role in neuronal development and neurite outgrowth, also supported by the evidence obtained in knockout (KO) or mutant LRRK2 neurons (Sepulveda et al., 2013). However, the specific roles of this fascinating protein still need to be fully defined. In this review, we will focus on the evidence pointing toward LRRK2-dependent modulation of striatal synaptic transmission, mitochondrial activity, and α-syn aggregation in both physiological and pathological conditions. Taking into account the frequency of LRRK2 gene abnormalities in familial and sporadic PD, the investigation of the molecular pathways influenced by mutant LRRK2 is particularly intriguing, and will potentially lead to the identification of effective neuroprotective therapies suitable for a large number of patients.

## From LRRK2 Gene Discovery to Development of Experimental Models

In 2002, the research team headed by Funayama performed a genome-wide linkage analysis of a Japanese family from Sagamihara region presenting familial parkinsonism with autosomal dominant transmission (Funayama et al., 2002). Patients from the “Sagamihara family” exhibited clinical features resembling classical PD, with an average onset of symptoms at 50 years of age (Funayama et al., 2002). A pathological study performed in this family showed that all cases had evidence of nigral degeneration at autopsy with varying amounts of LB pathology, ranging from completely undetected (at time of autopsy) to present and similar to conventional PD (Hasegawa et al., 2009). In one case, the pathological α-syn accumulation in glial cells was more widespread than what is usually observed in multiple systemic atrophy, an atypical parkinsonism (Hasegawa et al., 2009). The affected genomic locus was identified in the centromeric region of chromosome 12 (12p11.2-q13.1) and named PARK8 (Funayama et al., 2002). Interestingly, this haplotype was found not only in all the family members presenting a parkinsonian syndrome, but also in some unaffected carriers. This evidence suggested an incomplete penetrance of the mutation, with other genetic or environmental factors influencing the development of the disease (Funayama et al., 2002). A few years later, the linkage between PARK8 locus and PD was confirmed by a broad analysis of 21 Caucasian families with suspected autosomal dominant PD. This study showed the involvement of PARK8 in one family with German–Canadian kindred and one family from Western Nebraska (Zimprich et al., 2004b). The autoptic analysis of patients coming from these families confirmed the pleomorphic pathological picture of PARK8-related PD, ranging from a diffuse LB disease to a form of pure nigral degeneration (Zimprich et al., 2004a). Interestingly, the identification of PARK8 mutation in four Basque families, characterized by multiple cases of clinically typical PD with a mean age of 55 years at disease onset, suggested that this could be a commonly affected locus worldwide (Paisán-Ruíz et al., 2004; Paisàn-Ruìz et al., 2005). The specific gene was identified, thanks to two contemporary studies, published in the same *Journal*, and named LRRK2 or *dardarin* from the Basque term meaning “tremor” (Paisán-Ruíz et al., 2004; Zimprich et al., 2004a).

These advancements represented the beginning of LRRK2-centered research, which has led to the identification of 6 pathogenic LRRK2 mutations and more than 30 potentially pathogenic variants likely playing a key role in both familial and sporadic PD (Cookson, 2010; Monfrini and Di Fonzo, 2017). Among LRRK2 mutations, the one leading to the glycine-to-serine substitution G2019S in the LRRK2 protein was identified with unexpected high frequency (Di Fonzo et al., 2005; Gilks et al., 2005; Kachergus et al., 2005; Nichols et al., 2005). Indeed, the G2019S mutation was detected in approximately 5% to 6% of large familial European and American PD cohorts (Di Fonzo et al., 2005; Nichols et al., 2005) and in approximately 1% to 2% of sporadic PD from the United Kingdom (Gilks et al., 2005). Moreover, a very high frequency of G2019S mutation was identified in North African descent (up to 37%) and Ashkenazi Jewish (23%) familial and sporadic PD cases (Lesage et al., 2006; Ozelius et al., 2006). Overall, even taking into account the differences between various ethnicities, the G2019S LRRK2 mutation has arisen as the most frequent genetic determinant of PD (Monfrini and Di Fonzo, 2017). R1441 is the second most common pathogenic residue involved, with three known nonsynonymous (R1441C, R1441G, and R1441H) substitutions identified in several families worldwide (Puschmann, 2013; Monfrini and Di Fonzo, 2017). Of note, members of the Sagamihara family were found to carry a mutation in the I2020 residue of LRRK2 (I2020T), which is located in the kinase domain of the protein (Funayama et al., 2005).

Most LRRK2 mutations were found to be located within the catalytic core domains of LRRK2, specifically Roc-GTPase and kinase domains (Cookson, 2010; Benson et al., 2018; Outeiro et al., 2019). Indeed, the common site of mutation R1441 (G/C/H) is located in the Roc-GTPase domain, whereas G2019S mutation involves the kinase domain itself, increasing its activity by twofold to threefold (West et al., 2005; Greggio et al., 2006; Jaleel et al., 2007; Nichols et al., 2010; Steger et al., 2016). Of note, the activity of the Roc-GTPase domain is essential for intramolecular activation of LRRK2 serine–threonine kinase, as the binding with GTP leads to its autophosphorylation with the subsequent activation of downstream cell signaling pathways (Guo et al., 2007; Outeiro et al., 2019). Conversely, the hydrolysis of GTP is able to induce LRRK2 inactivation (Guo et al., 2007; Outeiro et al., 2019). Whether LRRK2 acts as a homodimer, interacting through its Roc-COR domains, or as a monomer is still under debate (Klein et al., 2009; Ito and Iwatsubo, 2012; Terheyden et al., 2015; Nixon-Abell et al., 2016). It has been hypothesized that the cytosolic protein could be mainly represented by a monomeric and kinase-inactive form, whereas the dimeric form is kinase-active and mainly found in association with cellular membranous structures (Berger et al., 2010; James et al., 2012). The evidence that LRRK2 mainly acts in a dimeric membrane-bound form suggested that its physiological functions could be primarily represented by the regulation of cellular processes involving membranes or vesicular dynamics. Moreover, considerig that pathogenic mutations were found to alter LRRK2’s active sites, the deregulation of the processes influenced by LRRK2 kinase activity could be crucial in PD development. Accordingly, several transgenic animal models, with different behavioral and neuropathological features, have been developed to unveil the pathophysiological consequences of abnormal LRRK2 function (Dawson et al., 2010; Blesa and Przedborski, 2014; Volta and Melrose, 2017).

The first transgenic mouse models were developed through the insertion of bacterial artificial chromosomes (BACs) carrying human or murine, mutant or wild-type LRRK2. These models were able to partially resemble the physiological specie-specific endogenous pattern of LRRK2 expression within the CNS, probably due to inappropriate regulation of gene expression induced by the insertion of exogenous human regulatory elements (Volta and Melrose, 2017). Overall, the BAC models expressing G2019S or R1441G/C exhibit mild abnormalities in striatal DAergic transmission, without significant nigral degeneration or LB accumulation (Li et al., 2009, 2010; Melrose et al., 2010; Sanchez et al., 2014; Walker et al., 2014; Beccano-Kelly et al., 2015; Volta et al., 2015; Sloan et al., 2016). From a behavioral point of view, BAC transgenic mice showed different phenotypes. Specifically, human BAC R1441G-LRRK2 mouse models showed a progressive and age-dependent hypokinesia reminiscent of PD, responsive to pharmacological treatments with L-DOPA (Li et al., 2009; Bichler et al., 2013), which could evolve to a state of immobility similar to late PD akinesia, as assessed through home cage activity analysis, open field, and cylinder tests (Li et al., 2009). Conversely, BAC human G2019S-LRRK2 animals were characterized by a paradoxical mild hyperactivity during young/juvenile age and a subsequent progressive mild motor impairment with late cognitive deficits, not fully resembling a PD-like behavior (Melrose et al., 2010; Volta et al., 2015). Other authors showed that rats expressing human G2019S and R1441C LRRK2 developed an age-dependent and L-DOPA-responsive motor impairment (Sloan et al., 2016). It should be highlighted that the evidence obtained from BAC LRRK2 models could have been limited by the utilization of different background strains, by the possibility that endogenous LRRK2 expression could influence the phenotype of the animals, and by the fact that BAC models are produced through the random insertion of human or murine transgene with variable integration site and copy number (Volta and Melrose, 2017). While the error in copy number can be low, if it does occur the expression level will change accordingly making comparisons harder (Chandler et al., 2007). Moreover, the discussed studies have mainly compared control mice to mice overexpressing either wild-type or mutant LRRK2, because comparisons among animals overexpressing different genetic LRRK2 variants could be altered by many confounding factors. Collectively, these aspects could represent potential limitations of BAC transgenic model use in studies aimed at investigating the pathological and functional consequences of a specific LRRK2 mutation.

A different genetic strategy to study the role of LRRK2 was represented by the overexpression of LRRK2 through complementary DNA (cDNA) under the control of specific promoters. With this technique, different research groups developed transgenic models overexpressing mutant or wild-type LRRK2 in the whole brain or selectively in DAergic neurons (Ramonet et al., 2011; Zhou et al., 2011; Chen et al., 2012; Maekawa et al., 2012; Chou et al., 2014; Liu et al., 2015; Weng et al., 2016). The investigation of these genetic models led to a wide variety of results. For instance, it has been shown that the expression of human G2019S LRRK2 in the whole brain, including SNpc, is accompanied by a progressive loss of tyrosine hydroxylase–positive (TH^+^) DAergic neurons with respect to nontransgenic littermates (Ramonet et al., 2011). Interestingly, the loss of TH^+^ nigral cells was paralleled by a reduction of Nissl^+^ nigral neurons, suggesting a neuronal degeneration, similar to that observed in LRRK2-related and idiopathic PD, rather than a loss of DAergic phenotype (Ramonet et al., 2011). This observation is further supported by the evidence that transgenic mice expressing human G2019S LRRK2 were characterized by a significant reduction in the number of SNpc DAergic neurons, whereas age-matched transgenic mice expressing human wild-type LRRK2 did not (Chen et al., 2012). Recently, a study investigating a tetracycline-inducible conditional transgenic mouse model, specifically expressing G2019S LRRK2 in DAergic neurons under the control of TH promoter, found an age- and kinase-dependent degeneration of neurons producing DA and norepinephrine (Xiong et al., 2018). Conversely, transgenic mice overexpressing R1441C LRRK2 were characterized by signs of neuronal suffering and cytopathological abnormalities such as enlarged vacuolar structures resembling autophagic vacuoles and condensed aggregated mitochondria in the cerebral cortex, without degeneration of the nigrostriatal DAergic pathway probably due to the observed lack of transgene expression in the SNpc of the investigated mice (Ramonet et al., 2011). Of note, the mentioned cortical cytopathological abnormalities were more pronounced in mice overexpressing G2019S LRRK2 (Ramonet et al., 2011). Interestingly, even if there were no signs of neuronal loss in the midbrain, R1441C transgenic mice displayed an impairment of locomotor activity, which was related to the observed cortical involvement (Ramonet et al., 2011). In line with the previous study, conditional transgenic mice that selectively expressed human R1441C LRRK2 in DAergic neurons, under the control of the endogenous murine ROSA26 promoter, displayed abnormalities only at the nuclear envelope of nigral cells, without evidence of neuronal loss (Tsika et al., 2014). Significant loss of SNpc DAergic neurons in R1441C transgenic mice was reported in a different study, in which the expression of human R1441C LRRK2 was controlled by CMV enhancer and a platelet-derived growth factor β promoter (Weng et al., 2016). This conflicting observation could rely on the utilization of different murine strains (C57BL/6J vs. FVB/N mice) with different susceptibility to neurodegeneration and different gene promoters influencing the temporal patterns and/or the levels of neuronal transgene expression (Tsika et al., 2014; Weng et al., 2016). Similarly, the analysis of striatal DA levels in these models revealed partially conflicting results. Some authors found no differences in striatal DA concentration in G2019S and R1441C LRRK2 mouse models compared to nontransgenic littermates (Ramonet et al., 2011), whereas others showed a reduction of evoked DA levels in the R1441C model compared to wild-type mice (Weng et al., 2016). Conversely, a reduction in striatal DA content was found in mice with conditional overexpression of human G2019S LRRK2, selectively expressing the transgene in midbrain DAergic neurons, with respect to nontransgenic littermates and transgenic mice expressing human wild-type LRRK2 (Liu et al., 2015).

It should be noted that the absence of significant nigral degeneration should not be strictly interpreted as a limitation. Indeed, these models can be useful to understand the pathogenetic events occurring in the disease phases preceding neurodegeneration, potentially unveiling the early stages of LRRK2-related PD progression. Accordingly, an increased presence of high-molecular-weight species of α-syn, indicative of aggregation, was found in the striatum of a tetracycline-inducible transgenic human G2019S LRRK2 mouse model, using a CAMKIIα promoter and conditionally expressing the transgene in the forebrain, compared to nontransgenic animals and animals expressing the double-mutant, kinase-dead, G2019S/D1994A LRRK2 as functionally negative control (Xiong et al., 2017). Of note, in G2019S LRRK2 transgenic mice, the striatal accumulation of insoluble α-syn was accompanied by the presence of subtle behavioral deficits compared to nontransgenic and kinase-dead mice, even if the authors did not find significant loss of DAergic neurons in the midbrain (Xiong et al., 2017).

Overall, the studies using BAC and cDNA models suggest that LRRK2 plays a crucial role in the modulation of the nigrostriatal pathway and specifically of striatal DA release. Moreover, it should be mentioned that LRRK2 KO mice were extensively investigated, showing no abnormalities in the DAergic striatal transmission (Tong et al., 2010; Herzig et al., 2011; Hinkle et al., 2012; Tozzi et al., 2018b). This observation suggests that: (i) the lack of LRRK2 could be balanced by compensatory mechanisms; or (ii) the abnormalities of the nigrostriatal pathway observed in overexpressing LRRK2 models are mediated by a gain of function of the protein. In this context, the investigation of genetically modified knock-in (KI) mice could offer a more informative background than BAC- or c-DNA–based models, focusing on the effects of specific mutations without the confounding factors represented by the overexpression of altered LRRK2 together with endogenous LRRK2. Various G2019S and R1441C/G KI models have been developed on different murine genetic backgrounds (as reviewed in Volta and Melrose, 2017). Thanks to the studies performed in these transgenic models, it has been hypothesized that LRRK2 can modulate mitochondrial activity and corticostriatal synaptic transmission in an age-dependent manner, during both physiological and pathological conditions (Beccano-Kelly et al., 2015; Yue et al., 2015; Matikainen-Ankney et al., 2018).

Altogether, transgenic LRRK2 models can display a wide range of pathological features and functional abnormalities in the nigrostriatal pathway, which could help to unravel the pathogenetic events taking place before the irreversible loss of DAergic midbrain neurons.

## LRRK2 Involvement in Striatal Synaptic Transmission

### Synaptogenesis and Synaptic Function

**Figure 1:**
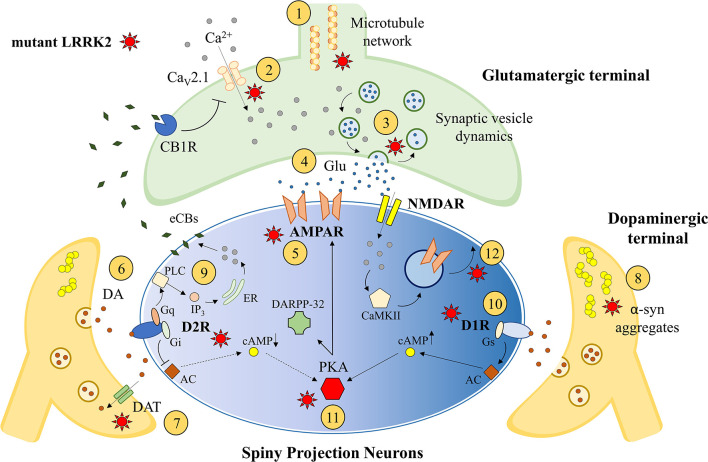
Striatal synaptic effects of leucine-rich repeatkinase 2 (LRRK2). LRRK2 is thought to influence striatal synaptogenesis interacting with cytoskeleton and microtubules ①. The modulation of presynaptic voltage-gated Ca^2+^ channels (Ca_V_2.1; ②), as well as the regulation of synaptic vesicle exocytosis, endocytosis, and recycling ③, could influence the release of glutamate (Glu) from the corticostriatal excitatory terminal ④. Moreover, the postsynaptic expression of glutamatergic AMPAR could be influenced by LRRK2 activity ⑤. LRRK2 could alter striatal DAergic transmission ⑥ inducing midbrain neuronal loss or DAergic terminals abnormalities, including abnormal DAT activity ⑦ and pathological α-synuclein aggregation ⑧. The postsynaptic expression and function of DA receptors can be influenced by mutant LRRK2, including altered D_2_R-dependent postsynaptic synthesis of endocannabinoids (eCBs; ⑨) and D_1_R expression/internalization ⑩. Finally, the dysfunction of PKA and DARPP32 pathways ⑪ and the impairment of intracellular AMPAR exocytosis ⑫ may result in altered synaptic long-term changes. AC, adenylate cyclase; CaMKII, Ca^2+^-calmodulin dependent protein kinase II; cAMP, 3′,5′-cyclic adenosine monophosphate; CB1R, cannabinoid receptor type 1; ER, endoplasmic reticulum; IP3, inositol trisphosphate; PLC, phospholipase C.

Possible LRRK2-induced abnormalities in corticostriatal synaptic transmission are suggested by the key role this protein plays in synaptogenesis and synaptic function (Esteves et al., 2014; Benson et al., 2018; Chen et al., 2018; Figure 1). Several studies, some of which investigating synaptic fraction preparations (Biskup et al., 2006; Piccoli et al., 2011), showed that LRRK2 is highly expressed in cerebral cortex and dorsal striatum compared to other brain areas (Taymans et al., 2006; Westerlund et al., 2008; Mandemakers et al., 2012; Davies et al., 2013; Giesert et al., 2013; West et al., 2014). Interestingly, the analysis of the LRRK2 gene expression patterns revealed a progressive temporal increase during *in vitro* neuronal development (Piccoli et al., 2011) and during postnatal development (Beccano-Kelly et al., 2014), reaching a maximum during the experience-dependent shaping of these connections (Benson et al., 2018). In line with this observation, some studies have shown that wild-type LRRK2 can modulate neurite outgrowth in developing neurons, because the LRRK2 KO was associated with abnormal elongation of neuronal processes (MacLeod et al., 2006; Parisiadou et al., 2009). Moreover, the G2019S LRRK2 mutation was found to be associated with a significant decrease in neurite length (Plowey et al., 2008). This effect, however, was found to be transient, overcome with time, and shown to be a function of velocity (Sepulveda et al., 2013). While the neurite outgrowth effect has been shown to be transient, it is no less important and illustrates another link to its potential in development of neurons.

The regulation of neurite outgrowth and synaptogenesis could rely on an LRRK2-dependent regulation of microtubule dynamics. Indeed, it has been reported that PD-causing mutations of LRRK2 can induce its abnormal binding to microtubules (Godena et al., 2014) and its aggregation into filamentous structures associated with the cytoskeleton in a well-ordered and periodic fashion (Kett et al., 2012).

Beyond synaptogenesis and cytoskeleton modulation, LRRK2 can exert additional roles at mature synaptic sites. Specifically, many studies have highlighted abnormal synaptic vesicle trafficking in transgenic LRRK2 models (Shin et al., 2008; Piccoli et al., 2011; Matta et al., 2012; Arranz et al., 2015; Belluzzi et al., 2016; Pan et al., 2017). LRRK2 was found to be associated with synaptic vesicle membranes where it could interact with vesicular proteins involved in exocytosis, endocytosis, and recycling dynamics, such as SNARE-complex proteins VAMP2, SNAP25, dynamin 1 and synaptophysin (Biskup et al., 2006; Piccoli et al., 2011, 2014). For instance, LRRK2 was found to interact with *N*-ethylmaleimide–sensitive fusion protein (NSF), which is a hexameric ATPase allowing the disassembling of SNARE proteins during synaptic vesicle exocytosis (Belluzzi et al., 2016). Mutations of LRRK2 associated with an increased kinase activity may impair synaptic vesicle dynamics through aberrant phosphorylation of NSF, potentially leading to altered neurotransmitter release (Belluzzi et al., 2016).

It may not be only exocytosis that is altered by mutant LRRK2, because vesicle endocytosis was also found to be abnormal in both G2019S and R1441C/G LRRK2 mutants (Shin et al., 2008; Pan et al., 2017; Nguyen and Krainc, 2018; Nguyen et al., 2019). In this context, the specific endocytic pathway modulated by LRRK2 has not yet been identified, but possible kinase substrates are represented by Rab proteins, Synaptojanin1, or EndoA, an evolutionary, conserved protein critically involved in synaptic vesicle endocytosis (Shin et al., 2008; Matta et al., 2012; Pan et al., 2017; Nguyen et al., 2019). Interestingly, G2019S LRRK2 mutation was found to be associated with impaired synaptic vesicle endocytosis in ventral midbrain neurons, including DAergic neurons, but not in neurons from the neocortex or the hippocampus, suggesting a region-specific effect (Pan et al., 2017). The same study showed that pharmacological inhibition of LRRK2 kinase activity rescued the observed endocytic defect in G2019S-expressing neurons, highlighting the involvement of the kinase domain in the modulation of synaptic dynamics (Pan et al., 2017). Lastly, it should be mentioned that LRRK2 could alter synaptic transmission not only by affecting exocytic/endocytic mechanisms, but also through interaction with voltage-gated calcium (Ca^2+^) channels (Ca_V_2.1 channels; Bedford et al., 2016). Indeed, LRRK2-dependent modulation of Ca^2+^ entrance at the presynaptic site could dramatically influence neurotransmitter vesicle release (Bedford et al., 2016). Furthermore, increased Ca^2+^ flux of this sort may influence Ca^2+^ stores in organelles affecting their function. Mitochondrial Ca^2+^ content, for instance, may influence mitochondrial membrane potential and ATP production (*via* dehydrogenase enzymes; Duchen, 2000; Denton, 2009). As mentioned earlier, ATP levels influence vesicle recycling and thus neurotransmitter release (Belluzzi et al., 2016), providing another route for altered neurotransmission. Overall, accumulating evidence suggests that LRRK2 PD-linked mutations could alter synaptic vesicle trafficking, potentially leading to abnormalities in striatal synaptic transmission, as well as to toxic effects contributing to the neurodegenerative process leading to PD (Nguyen et al., 2019).

### Glutamatergic Synaptic Transmission

The disruption of cortical glutamatergic inputs to the nucleus striatum could result in a severe alteration of the whole basal ganglia network. As such, possible alterations of striatal glutamatergic neurotransmission have been investigated in several KI LRRK2 experimental models. As discussed, the levels of LRRK2 are comparatively higher in both striatal and cortical regions (Melrose et al., 2006, 2007; Lee et al., 2010), making these areas worthy of investigation. Increase of spontaneous glutamatergic activity was observed in striatal neurons of G2019S LRRK2 KI mice during the postnatal period (Matikainen-Ankney et al., 2016; Volta et al., 2017), as well as in G2019S LRRK2 KI cortical neuronal cultures (Beccano-Kelly et al., 2014). Specifically, glutamate release was found to be markedly elevated in 3-week-old G2019S KI cortical neuronal cultures, without changes in synapse density. This observation suggested that the enhanced release could depend on increased vesicle release probability due to altered presynaptic regulatory protein profile (Beccano-Kelly et al., 2014; Piccoli et al., 2014). In acute corticostriatal slices, obtained from less than 1-month postnatal G2019S LRRK2 KI mice, spontaneous glutamatergic activity onto SPNs was significantly increased, both in the direct and indirect basal ganglia pathway (Matikainen-Ankney et al., 2016; Volta et al., 2017). The acute *in vitro* exposure to LRRK2 kinase inhibitors, as well as the isolation of the striatum from the overlying neocortex, were able to normalize the excitatory transmission in G2019S mutants, supporting an LRRK2 kinase-dependent alteration of corticostriatal function (Matikainen-Ankney et al., 2016). The hypothesis that LRRK2 kinase hyperactivity is required to induce synaptic changes is further supported by the evidence that subtle synaptic abnormalities were found in wild-type LRRK2 overexpressing (~3×) neurons (Beccano-Kelly et al., 2014).

The effects of G2019S LRRK2 mutation on glutamatergic transmission appear to be age-dependent, prominent in young mice and progressively declining with age (Matikainen-Ankney et al., 2016; Volta et al., 2017). This is in line with the hypothesized involvement of LRRK2 in shaping neural connections during the postnatal development of striatal circuits, with potential permanent consequences (Matikainen-Ankney et al., 2016). The age-dependent effects of LRRK2 kinase hyperactivity on synaptic transmission are paralleled by the presence of behavioral abnormalities in young mice, such as an increased spontaneous exploration, which progressively normalize with time (Volta et al., 2017). Accordingly, different authors showed normal spontaneous glutamatergic transmission in adult LRRK2 KI mice (Matikainen-Ankney et al., 2016; Volta et al., 2017; Tozzi et al., 2018a) and adult BAC mice overexpressing human wild-type LRRK2 (Beccano-Kelly et al., 2015) compared to nontransgenic animals. Of interest, it has been proposed that the effects of LRRK2 kinase hyperactivity on glutamatergic corticostriatal transmission could still be present but more subtle during the adult age, unveiled only during specific tasks or by the activation of the DAergic receptors (Beccano-Kelly et al., 2015; Tozzi et al., 2018a). Specifically, the pharmacological stimulation of DA D_2_R was able to induce an enhanced reduction of glutamatergic transmission in 6-month-old G2019S LRRK2 KI mice compared to age-matched wild-type mice, and this effect was hypothesized to be dependent on a greater release of retrograde messengers from the SPNs (Tozzi et al., 2018a). It should be noted that the acute *in vitro* inhibition of LRRK2 kinase was not able to reverse the observed effect of D_2_R activation, suggesting that the constitutive LRRK2 kinase activation in striatal SPNs could permanently shape striatal connections (Tozzi et al., 2018a). The involvement of D_2_R in the LRRK2-dependent modulation of excitatory transmission could be an intriguing field of research, because the increased glutamate release observed in young G2019S KI mice was not influenced by the pharmacological agonism of D_2_R (Volta et al., 2017). Thus, the loss of the D_2_R-dependent physiological inhibitory effect on striatal excitatory transmission may contribute to the enhanced glutamate release observed in young G2019S KI mice, with a subsequent age-dependent recovery leading to enhanced inhibition in adult mice.

Lastly, it has been shown that glutamatergic transmission could be influenced by LRRK2-induced changes to postsynaptic glutamatergic receptors, such as AMPA receptor (AMPAR) subunit expression. Indeed, an increase in amplitude of spontaneous excitatory postsynaptic currents was shown in LRRK2 KO mice compared to wild-type animals, potentially due to increased expression of GluR1 AMPAR subunit at the synaptic site (Parisiadou et al., 2014). Moreover, mice expressing G2019S LRRK2 KI mutation lacked functional calcium-permeable AMPARs in SPNs of the nucleus accumbens (Matikainen-Ankney et al., 2018).

### Dopaminergic Synaptic Transmission

As previously discussed, the various existing transgenic LRRK2 experimental models are characterized by different degrees of DA depletion within the nucleus striatum. Considering that LRRK2 transgenic models investigated so far are characterized by various levels and spatial/temporal patterns of LRRK2 expression (mutant or wild-type), it is difficult to conclude if LRRK2-related PD is associated with a primary damage of DAergic cells in the midbrain or with an isolated dysfunction of striatal DAergic terminals. Indeed, transgenic LRRK2 models based on the use of the cDNA usually displayed nigral DAergic neuronal loss (Ramonet et al., 2011; Chen et al., 2012; Weng et al., 2016; Xiong et al., 2018), whereas the transgenic BAC mice were not characterized by neurodegenerative features in the midbrain (Li et al., 2009, 2010; Melrose et al., 2010; Sanchez et al., 2014; Walker et al., 2014; Beccano-Kelly et al., 2015; Volta et al., 2015; Sloan et al., 2016).

Focusing on the effects of increased LRRK2 activity upon DA transmission in mutants, an age-dependent reduction of basal striatal extracellular DA levels has been shown in G2019S KI mice, which was hypothesized to be dependent on a latent impairment of synaptic DA release (Yue et al., 2015). A subsequent study suggested that striatal DA loss in G2019S KI mice could represent the consequence of an altered regulation of DA release and/or nigral burst firing patterns *in vivo*, rather than impaired single synapse release or DA transporter (DAT) activity (Volta et al., 2017). Moreover, slices from young G2019S KI mice displayed enhanced DA release upon repeated stimulation compared to wild-type animals. This effect was no longer evident in old animals, suggesting that DAergic transmission could be modulated by LRRK2 in an age-dependent manner similarly to glutamatergic transmission (Volta et al., 2017). Considering these results, the authors suggested that one possible explanation would be that the hyperactivation of LRRK2 could induce a premature aging of DAergic terminals (Volta et al., 2017). Another such hypothesis would be the acute-cum-chronic compensation, which occurs due to D_2_R insensitivity at young ages and results in an unsustainable situation and synaptic stress.

The hypothesis of a specific DAergic terminal vulnerability is further supported by the results obtained by other groups, showing that G2019S mutation is associated with lower DA striatal levels in old mice (Tozzi et al., 2018b) and is able to progressively alter DAT activity together with α-syn accumulation at striatal DAergic terminals (Longo et al., 2017). Accordingly, BAC transgenic mice overexpressing G2019S LRRK2 showed age-dependent decrease of striatal DA content, release, and uptake, with possible selective DAergic terminal damage because no nigral cell loss was detected (Li et al., 2010). Finally, a transgenic model selectively expressing the G2019S LRRK2 in midbrain DAergic neurons displayed no substantial SNpc neuronal loss (Liu et al., 2015). However, it was possible to detect a reduction of striatal DA content and release, coincident with the degeneration of DAergic axonal terminals and with the reduction of the enzymatic machinery responsible for DA synthesis, transport, and degradation (Liu et al., 2015). Overall, despite the previously discussed limitations of existing LRRK2 experimental models, accumulating evidence suggests that the expression of mutant G2019S LRRK2 could induce a selective damage of DAergic axon terminals in the nucleus striatum, potentially preceding midbrain neuronal loss. This is further supported by the observation that G2019S LRRK2 expressing mice were characterized by early-phase dysfunction of SNpc DAergic neurons, including a reduction in striatal evoked DA release, several months before the irreversible degeneration of these cells (Chou et al., 2014), as observed also in BAC mice overexpressing human wild-type LRRK2 (Beccano-Kelly et al., 2015).

Mutant LRRK2 does not alter only DAergic projections, as reported by different studies describing the presence of abnormal DA receptor expression and function in transgenic LRRK2 models. For instance, it has been demonstrated that transfection of SH-SY5Y cells with G2019S or R1441G LRRK2 increased the expression of DA D_1_R, an effect confirmed by Western blot analysis of striatal membrane fractions obtained from transgenic mice overexpressing G2019S LRRK2, showing increased D_1_R expression with respect to nontransgenic animals (Migheli et al., 2013). In addition, mutant G2019S LRRK2 could impair the internalization of D_1_R, which should take place after its sustained activation, prolonging the activation of its signaling transduction pathway and increasing intraneuronal production of cAMP (Rassu et al., 2017). The D_1_R transduction pathway could be influenced by LRRK2 activity itself, because the protein kinase A (PKA)-dependent phosphorylation of synaptic AMPAR subunit GluR1 was abnormally enhanced after treatment with a D_1_R agonist in a mouse model lacking LRRK2 (Parisiadou et al., 2014). Collectively, these effects could contribute to the abnormal striatal synaptogenesis and transmission observed in LRRK2 transgenic models. Moreover, G2019S LRRK2 was also able to influence the physiological turnover of D_2_R by decreasing the rate of its trafficking from the Golgi complex to the cell membrane (Rassu et al., 2017). In apparent contrast with this observation, other studies highlighted the presence of unaltered D_2_R expression in transgenic LRRK2 mouse models (Li et al., 2010; Melrose et al., 2010).

Overall, it has been hypothesized that LRRK2 overexpression could influence D_2_R surface expression, with variations depending on the analyzed model, whereas the mutations leading to LRRK2 hyperactivation could influence the downstream D_2_R signaling pathway (Volta and Melrose, 2017). In line with this hypothesis, abnormal D_2_R function was identified in young G2019S KI mice, where the physiological D_2_R-dependent negative regulation of glutamatergic transmission was absent (Volta et al., 2017), whereas an increased inhibitory effect following D_2_R activation was found in adult G2019S KI mice (Tozzi et al., 2018a). In this last work, it has been shown that the enhanced inhibition of excitatory transmission was mediated by the postsynaptic release of endocannabinoids (eCBs), produced after phospholipase C (PLC) activation, which act as retrograde messengers on the presynaptic cannabinoid receptor type 1 (CB1R; Tozzi et al., 2018a). Because the function of CB1R was not itself altered, an increased activation of the D_2_R/PLC/eCBs pathway in SPNs of adult transgenic KI mice has been hypothesized (Tozzi et al., 2018a). Lastly, another interesting report has shown the presence of altered D_2_R signaling in a transgenic model overexpressing LRRK2 (Beccano-Kelly et al., 2015). Specifically, the authors showed an alteration of another postsynaptic D_2_R-dependent transduction pathway, involving the PKA-regulated phosphoprotein DARPP-32 (Beccano-Kelly et al., 2015).

The effects of LRRK2 activity on DA receptor expression and function deserve further investigation. Considering that LRRK2 is poorly expressed in DAergic nigral cells but highly expressed in the striatum (Melrose et al., 2006, 2007; Lee et al., 2010), abnormalities of striatal DA transmission could be a result of postsynaptic rather than presynaptic alterations. In this scenario, a more in-depth characterization of the LRRK2-depedent modulation of DA receptors could help in understanding the mechanisms leading to the striatal synaptic dysfunction occurring in PD (Calabresi et al., 2007, 2014; Schirinzi et al., 2016).

### Striatal Synaptic Plasticity

Considering that LRRK2 activity influences both glutamatergic and DAergic striatal synaptic transmission, it is reasonable to hypothesize the presence of alterations of synaptic long-lasting changes. Interestingly, recent work has shown that both D_1_R- and D_2_R-expressing SPNs were unable to express synaptic long-term potentiation (LTP) at corticostriatal synapses in G2019S LRRK2 KI mice, probably because of an LRRK2-dependent impairment of AMPAR trafficking (Matikainen-Ankney et al., 2018). Of note, D_2_R-expressing SPNs exhibited synaptic long-term depression (LTD) after the stimulation protocol able to induce LTP in wild-type mice (Matikainen-Ankney et al., 2018). This observation is in line with the previously discussed enhancement of D_2_R-dependent eCB release observed in G2019S LRRK2 mice (Tozzi et al., 2018a). Moreover, it should be considered that the activation of DA D_2_Rs normally exerts a negative control on the induction of *N*-methyl-D-aspartate receptor (NMDAR)-dependent LTP, and the induction of LTD is thought to require a weaker DAergic input because of the higher affinity of D_2_R for DA compared to D_1_R (Jaber et al., 1996; Calabresi et al., 2007). The presence of reduced DA levels in the striatum of LRRK2 G2019S KI mice (Liu et al., 2015; Tozzi et al., 2018b), together with an enhanced D_2_R signaling, could favor the induction of an eCB-dependent LTD in D_2_R-expressing SPNs. This hypothesis deserves further investigations, since other authors have shown an impairment of striatal LTD induction, together with reduced evoked striatal DA release, in transgenic mice overexpressing human G2019S LRRK2 (Chou et al., 2014).

In physiological conditions, the molecular mechanisms leading to LTP induction involve the activation of Ca^2+^-calmodulin-dependent protein kinase II (CaMKII), which can increase the number of AMPARs expressed at the postsynaptic membrane through the exocytosis of intracellular vesicular AMPAR pools or through lateral diffusion of extrasynaptic receptors (Choquet and Triller, 2003; Malenka and Bear, 2004; Opazo et al., 2012; Nicoll, 2017). In this scenario, an LRRK2-induced alteration of vesicle trafficking (discussed earlier) could play a critical role. LRRK2 could influence the LTP-dependent AMPAR synaptic expression through interaction with Rab8a (Steger et al., 2016), a small vesicular transport protein acting as a critical component of the molecular pathway leading to AMPARs insertion into synapses (Gerges et al., 2004). Another possible mechanism explaining the aberrant synaptic plasticity is the dysregulation of PKA/DARPP32 pathway. In the striatum, D_1_R and D_2_R exert opposite effects on PKA activity, stimulating and inhibiting its function (Calabresi et al., 2007). Once activated, PKA plays a key role in the modulation of LTP and LTD induction, mediating the synaptic incorporation of AMPARs through the phosphorylation of GluR4 and GluR1 subunits (Esteban et al., 2003) and activating the DA- and cAMP-regulated DARPP32 protein, which acts as an inhibitor of protein phosphatase 1 (Greengard et al., 1999; Calabresi et al., 2007). Interestingly, as previously discussed, LRRK2 interacts with both PKA and DARPP32 (Parisiadou et al., 2014; Beccano-Kelly et al., 2015; Greggio et al., 2017; Tozzi et al., 2018b) and a better understanding of the effects induced by LRRK2 mutants on these proteins could explain the observed alterations of striatal synaptic plasticity.

Of note, the effects of LRRK2 kinase activity on synaptic plasticity could go beyond corticostriatal connections, because the induction of synaptic LTD was impaired in the hippocampus of BAC transgenic mice overexpressing G2019S LRRK2 (Sweet et al., 2015). Also in this case, an impairment of AMPAR trafficking behind the synaptic defect was hypothesized, because its internalization during LTD induction could be impaired by LRRK2 hyperactivation (Sweet et al., 2015). Collectively, the mechanisms leading to synaptic long-term changes could be disrupted in the presence of abnormal LRRK2 kinase activity, and this synaptic dysfunction could take place long before the progressive loss of DAergic nigral cells, accompanied by the presence of clinical symptoms (dystonia, goal-directed movement dysfunction), which are thought to rely on such functions (Mink, 2018). The identification of the molecular pathways involved in this process could unveil new therapeutic strategies aimed at preserving neural network activity earlier in PD progression.

## LRRK2 Involvement in Mitochondrial Function

Mitochondrial dysfunction is considered a crucial pathogenic mechanism in the neurodegenerative process leading to PD (Schapira, 2007; Bose and Beal, 2016). Epidemiological studies highlighted a possible association between PD development and exposure to environmental toxic agents targeting mitochondrial activity, such as pesticides or herbicides (Kalia and Lang, 2015). Moreover, the recreational use of a meperidine analog, 1-methyl-4-phenyl-4-propionoxypiperidine, was associated in some individuals with the development of a parkinsonian syndrome (Langston et al., 1983; Ballard et al., 1985). The pathogenesis of this syndrome was found to be caused by the presence of a contaminant molecule, 1-methyl-4-phenyl-1,2,3,6-tetrahydropyridine (MPTP), which could be converted in a compound targeting mitochondrial respiratory chain complex I (Nicklas et al., 1985). Subsequently, the activity of mitochondrial complex I was found to be reduced in several tissues isolated from PD patients (Schapira, 2007; Bose and Beal, 2016), and mitochondrial complex I inhibitors, such as MPTP or rotenone, were found to be able to lead to the somewhat specific death of catecholaminergic neurons including nigral DA cells and have been widely employed to induce experimental PD models (Cicchetti et al., 2009; Bezard and Przedborski, 2011).

**Figure 2:**
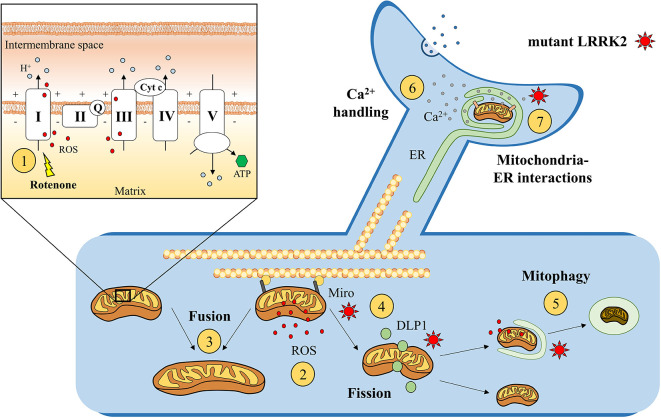
Suggested mitochondrial effects of mutant LRRK2. Enhanced susceptibility to rotenone, a mitochondrial chain complex I inhibitor, was found in genetic LRRK2 experimental models ①. This increased susceptibility to mitochondrial dysfunction could impair ATP formation and enhance reactive oxygen species (ROS) production ② after the exposure to endogenous or exogenous mitochondrial stressors. Moreover, LRRK2 is hypothesized to alter mitochondrial fusion ③ and fission ④ process, by interacting with mitochondrial docking proteins, such as Miro, or dynamin-like protein 1 (DLP1). Abnormally active LRRK2 could also impair the removal of damaged mitochondria through lysosomal-dependent mitophagy ⑤ and alter mitochondrial Ca^2+^ buffering activity ⑥ at mitochondria–ER interactions ⑦. On the upper left, simplified representation of the mitochondrial electron transport chain (including mitochondrial complexes I, II, III, IV, and V; coenzyme Q; and cytochrome c).

The discovery and the study of the genetic abnormalities linked to PD further supported the importance of mitochondrial dysfunction in the pathogenetic process leading to the development of the disease. Many of the proteins encoded by genes causing recessive, atypical forms of PD, such as parkin, PINK1, and DJ-1, are involved in mitochondrial homeostatic processes (Lin and Beal, 2006; McCoy and Cookson, 2012; Kalia and Lang, 2015; Bose and Beal, 2016). The investigation of the pathophysiological consequences of LRRK2 mutations also unveiled a mitochondrial regulatory role for this protein (Esteves et al., 2014; Singh et al., 2019). Indeed, the presence of LRRK2 mutations has been linked to abnormalities in mitochondrial ATP and reactive oxygen species (ROS) production, mitochondrial fusion and fission dynamics, mitophagy, mitochondrial DNA (mtDNA) damage, and calcium homeostasis (Figure 2). For instance, analysis of mitochondrial function and morphology in skin biopsies obtained from LRRK2 mutant patients revealed the presence of altered mitochondrial membrane potential, reduced ATP levels, mitochondrial elongation, and increased mitochondrial interconnectivity in the G2019S mutation carriers (Mortiboys et al., 2010).

A mitochondrial regulatory role for LRRK2 is also supported by the evidence that, in LRRK2 overexpressing models, approximately 10% of the protein was found in the mitochondrial cell fractions, with immunohistochemical and biochemical studies suggesting a mitochondrial localization (West et al., 2005; Biskup et al., 2006). The preferential association of LRRK2 with a variety of cellular membrane and vesicular structures suggests an affinity of LRRK2 for lipids or lipid-associated proteins and a potential localization in mitochondrial outer membrane (West et al., 2005; Biskup et al., 2006). Such localization could influence mitochondrial fusion and fission processes, crucial for the maintenance of a functional mitochondrial network along the neuron and the axon (Cho et al., 2010; Su et al., 2010; Bertholet et al., 2016). It should be noted that the large amount of evidence for LRRK2 effects on mitochondria is not matched by a corresponding amount of data supporting a physical interaction (from Berwick et al., 2019). Indeed, a direct association between LRRK2 and mitochondrial membranes was not confirmed by subsequent studies, and the utilization of tagged LRRK2 suggested an association with different cellular structures such as endosomes/endoplasmic reticulum (ER; Gómez-Suaga et al., 2014; Schreij et al., 2015).

The fusion and fission of neuronal mitochondria are highly regulated processes, which could be disrupted by mutant LRRK2. Indeed, aged LRRK2 G2019S KI mice were characterized by profound mitochondrial abnormalities in the striatum, consistent with arrested fission (Yue et al., 2015). Other authors suggested that LRRK2 regulates mitochondrial dynamics through direct interaction with a fission dynamin-like protein 1 (DLP1 or DRP1), because LRRK2 overexpression was associated with mitochondrial fragmentation together with increased DLP1 expression (Niu et al., 2012; Wang et al., 2012). The role of DLP1 in mitochondrial fission is well established (Chang and Blackstone, 2010), and considering that mitochondrial fragmentation is thought to precede the elimination of dysfunctional mitochondria, a physiological DLP1 activity could facilitate mitochondrial elimination after a toxic stimulus (Arnoult et al., 2005). Disruption of this process could lead to abnormal mitochondrial fragmentation in physiological conditions and/or reduced mitochondrial elimination after exposure to environmental toxic agents. Both these processes could facilitate the development of PD. Interestingly, abnormal LRRK2 kinase activity was able to alter the DLP1-regulated biological processes (Wang et al., 2012). Specifically, LRRK2 G2019S mutation was found to enhance the translocation of DLP1 from cytosol to mitochondria leading to enhanced mitochondrial fission (Niu et al., 2012). In line with this hypothesis, fragmented and dysfunctional mitochondria were found in fibroblasts obtained from G2019S carriers (Grünewald et al., 2014), and treatment with a pharmacological inhibitor of DLP1 was able to reduce mitochondrial fragmentation in LRRK2 G2019S-expressing cells and PD patient fibroblasts (Su and Qi, 2013). Overall, the functional interactions between LRRK2 and DLP1 seem to be involved in the regulation of mitochondrial dynamics, as further suggested by the evidence that a recently identified LRRK2 variant, E193K, was able to alter the possible LRRK2/DLP1 binding, leading to an abnormal mitochondrial fission after a metabolic insult (Perez Carrion et al., 2018).

Other groups also found alterations in mitochondrial reaction to toxic agents in LRRK2 models. Indeed, it has been shown that G2019S LRRK2-overexpressing SHSY5Ycells were characterized by abnormally highly fragmented mitochondrial network after exposure to rotenone, a mitochondrial complex I inhibitor (Tozzi et al., 2018b). In this case, the potential involvement of DLP1 was not investigated, but the pharmacological activation of D_2_R was able to counteract the abnormal mitochondrial fragmentation, suggesting the involvement of additional pathways linking LRRK2 activity and mitochondrial dynamics that deserve further investigation (Tozzi et al., 2018b). In this context, it should be noted that increased mitochondrial fragmentation after toxic stimuli could represent a neuronal attempt to remove dysfunctional mitochondria, thus meaning an enhanced mitochondrial vulnerability to environmental toxic injuries in the presence of abnormal LRRK2 kinase activity. Moreover, mitochondria that underwent fission process should be subsequently eliminated through autophagic mechanisms, but an alteration at this level could increase the content of uncleared fragmented mitochondria. For now, it is difficult to have a single answer to these questions because available studies support both hypotheses.

An investigation performed in *Caenorhabditis elegans* showed that human wild-type LRRK2 reduced the toxic effect of mitochondrial toxins, such as rotenone or paraquat, but this protective effect was lost in G2019S LRRK2-expressing nematodes, with a rapid loss of DAergic markers (DAT:GFP fluorescence and dopamine levels; Saha et al., 2009). Indeed, increased susceptibility to rotenone-induced toxicity has been described in transgenic *Drosophila*-expressing mutant LRRK2, including G2019S (Ng et al., 2009). A similar enhanced cellular susceptibility to mitochondrial dysfunction was described in neural cells generated from induced pluripotent stem cells (iPSCs) obtained from LRRK2 PD patients (Cooper et al., 2012) and DAergic neurons derived from iPSCs of patients carrying the G2019S mutation (Nguyen et al., 2011). Accordingly, transgenic G2019S mice seem to be more vulnerable to the detrimental effect of mitochondrial toxins (Tozzi et al., 2018b). Specifically, it has been shown that the neurotoxic effect induced by rotenone exposure was enhanced in corticostriatal slices obtained from G2019S KI mice, relative to wild-type, LRRK2 kinase-dead, and LRRK2 KO mice, suggesting that the sustained activation of LRRK2 kinase domain was involved (Tozzi et al., 2018b). Furthermore, the rotenone-dependent reduction of cellular ATP synthesis, associated with increased ROS production, was significantly enhanced in SHSY5Y cells overexpressing G2019S LRRK2 compared to control cells (Tozzi et al., 2018b). Of note, the pharmacological activation of D_2_R reduced rotenone toxicity in G2019S LRRK2 KI mice, with potential involvement of the cAMP/PKA pathway because the pharmacological inhibition of PKA was able to mimic the D_2_R-dependent protective effect in G2019S KI mice, whereas the exposure to a cAMP analog enhanced rotenone toxicity in the striatum of wild-type mice (Tozzi et al., 2018b). In this scenario, the possible involvement of PKA pathway in mitochondrial homeostasis should be further investigated to be better understood (Valsecchi et al., 2013; Di Benedetto et al., 2018), considering that PKA-dependent phosphorylation of mitochondrial proteins may enhance mitochondrial ROS production (Prabu et al., 2006; Fang et al., 2007). Interestingly, the induced expression in cultured cortical neurons of both wild-type and G2019S LRRK2 was associated with increased cellular ROS production, an effect not seen with the kinase-dead mutant LRRK2 (Niu et al., 2012), and LRRK2 kinase hyperactivity could reduce the antioxidant mitochondrial defense through interaction with peroxiredoxin-3, the most important mitochondrial scavenger of hydrogen peroxide (Angeles et al., 2014). The presence of increased ROS production could trigger a vicious cycle through mtDNA damage, leading to irreversible mitochondrial dysfunction. Accordingly, LRRK2 G2019S patient-derived lymphoblastoid cell lines (LCLs) and iPSC-derived neural cells exhibited increased mtDNA damage (Sanders et al., 2014; Howlett et al., 2017), and treatment with an LRRK2 kinase inhibitor (Howlett et al., 2017) or the zinc finger nuclease-mediated gene correction of G2019S mutation (Sanders et al., 2014) was able to prevent or to restore it.

During physiological conditions, dysfunctional and damaged mitochondria are removed through lysosomal-dependent mitophagy. Several studies suggested that LRRK2 could modulate this cellular process (Ferree et al., 2012; Beilina et al., 2014; Schapansky et al., 2014; Hsieh et al., 2016; Wallings et al., 2019). Experiments through protein–protein interaction arrays revealed a possible link among LRRK2 and BCL2-associated athanogene 5, Rab7L1 (RAB7, member RAS oncogene family-like 1), and cyclin-G-associated kinase, all of which are involved in the autophagy–lysosome system (Beilina et al., 2014). A role in the regulation of autophagy was suggested by the evidence that silencing endogenous LRRK2 expression, or its kinase activity inhibition, resulted in deficits of the autophagic processes in immune cells (Schapansky et al., 2014), as well as by a transcriptome analysis of human brain, human blood cells, and *C. elegans* expressing human wild-type LRRK2 (Ferree et al., 2012). LRRK2 was shown to significantly contribute to autophagosome-lysosome fusion and lysosomal pH. This is achieved *via* direct binding of LRRK2 to the vacuolar-type H^+^-ATPase pump a1 (Wallings et al., 2019). Overall, it can be hypothesized that some of LRRK2 PD–related mutations may alter the neuronal ability to degrade damaged intracellular organelles. Interestingly, a possible molecular mechanism linking LRRK2 and mitophagy has been suggested by a work showing in iPSC-derived neurons that wild-type LRRK2 promotes the removal of a mitochondrial docking protein, Miro, as an early step in dysfunctional mitochondria clearance (Hsieh et al., 2016). The presence of G2019S mutation disrupted this physiological LRRK2 function, delaying the arrest of damaged mitochondria with subsequent impairment of mitophagy (Hsieh et al., 2016). Of note, Miro degradation and mitochondrial motility were also found to be impaired in fibroblasts obtained from sporadic PD patients (Hsieh et al., 2016), suggesting that this pathway could be commonly involved during the development of familial and idiopathic PD. Thus, the emerging picture seems extremely complex, with LRRK2 potentially influencing mitochondrial chain complex activity, susceptibility to oxidative stress, and mitochondrial removal pathways. Overall, the presence of LRRK2 mutations could influence the ability of DAergic neurons to cope with exposure to environmental or endogenous mitochondrial stressors, acting as a strong predisposing factor for PD. This observation could explain the frequency of LRRK2 abnormalities in familial and sporadic PD, thus increasing the potential impact of LRRK2-centered neuroprotective strategies.

As a concluding remark, it should be considered that LRRK2 could also alter physiological mitochondrial Ca^2+^ buffering activity. The entrance of Ca^2+^ into the mitochondrial matrix through mitochondrial Ca^2+^ uniporter is made possible by the electrochemical proton gradient created by the electron transport chain. Thanks to this property, mitochondria can dynamically uptake and release Ca^2+^, influencing the concentration of this ion in the whole cellular cytosol or in a specific subcompartment, such as presynaptic and postsynaptic terminals (Rizzuto et al., 2012). Dysregulation of this process could affect neuronal synaptic transmission *via* microdomain Ca^2+^ release and/or trigger cellular death, through the apoptotic or necrotic pathways. In this context, it has been shown that murine cortical neurons expressing mutant G2019S or R1441C LRRK2 were characterized by neuronal Ca^2+^ imbalance (Cherra et al., 2013). Also, LRRK2 G2019S iPSC-derived sensory neurons displayed altered Ca^2+^ dynamics, observed through live-cell Ca^2+^ imaging, which was counteracted by LRRK2 inhibitors (Schwab and Ebert, 2015).

Interestingly, in order to facilitate Ca^2+^ buffering, it has been shown that mitochondria are frequently located in proximity of specific cellular microdomains with local high Ca^2+^ concentration, such as the synaptic terminals, Ca^2+^ channels at the plasma membrane (David et al., 1998; Glitsch et al., 2002; Young et al., 2008), and the ER, with which the mitochondria closely interact (Rizzuto et al., 1998; Csordás et al., 2006, 2010). In this context, the structurally tethered ER–mitochondria interactions, named mitochondria-associated membranes (MAMs), can facilitate Ca^2+^ exchange and regulate local Ca^2+^ concentration, influencing various cellular processes including ATP production, autophagy, apoptosis, and synaptic transmission when located at the presynaptic sites (Simmen et al., 2010; Rizzuto et al., 2012; Rowland and Voeltz, 2012; Hamasaki et al., 2013; Kornmann, 2013; Marchi et al., 2014; Devine and Kittler, 2018). A possible structural and/or functional disruption of the MAMs is thought to occur during the development of various neurodegenerative diseases, including PD (Paillusson et al., 2016; Devine and Kittler, 2018). Interestingly, a recent publication showed that LRRK2 is involved in the regulation of ER–mitochondria interactions, with the evidence that the G2019S LRRK2 mutation could lead to the ubiquitin-mediated degradation of ER–mitochondrial tethering proteins (Toyofuku et al., 2020). Of note, it has been shown that also α-syn can localize at the level of MAMs, and its genetic abnormalities are associated with reduced mitochondria–ER apposition and abnormal Ca^2+^ exchange (Guardia-Laguarta et al., 2014; Paillusson et al., 2017). Overall, mitochondria–ER interactions represent an interesting avenue to be investigated, potentially unveiling new molecular pathogenic pathways linking LRRK2, α-syn, and mitochondrial homeostasis.

## LRRK2 and α-Synuclein Aggregation

The investigation of the molecular pathways linking LRRK2 and α-syn has attracted a lot of attention (Esteves et al., 2014; Schapansky et al., 2015; Cresto et al., 2019; Outeiro et al., 2019). A possible role for LRRK2 in the formation of abnormally folded α-syn aggregates was suggested by histopathological studies showing that LRRK2 could be found in the context of LBs. Specifically, immunohistochemical analysis of brain samples obtained from patients with confirmed PD and LB dementia revealed that 20% to 100% (mean, 60%) of α-syn–positive LBs contained LRRK2 (Perry et al., 2008). Interestingly, other authors showed that the presence of LRRK2 in the core of LBs was higher in the SNpc than in the locus coeruleus of brains obtained from sporadic PD patients, but the percentage of LBs with detectable LRRK2 was significantly higher in both the brain areas of patients carrying the G2019S LRRK2 mutation (Vitte et al., 2010). Accordingly, it has been shown that LRRK2 levels are positively correlated to pathological α-syn aggregation in the affected brain regions, colocalizing with neurons and LBs (Guerreiro et al., 2013).

Other clues on the possible relationship between LRRK2 and α-syn have been given by preclinical studies, highlighting molecular interactions between the two proteins in a cell culture model of α-syn inclusion formation (Guerreiro et al., 2013). Moreover, LRRK2 overexpression significantly accelerated the progression of α-syn aggregation in PD-related A53T SNCA transgenic mice, whereas the genetic ablation of LRRK2 was able to delay it (Lin et al., 2009).

It has been suggested that the abnormal LRRK2-induced α-syn aggregation and somatic accumulation could rely on altered microtubule dynamics and ubiquitin–proteasome pathway activity (Lin et al., 2009), which is linked to pathological α-syn expression (Bentea et al., 2015). Interestingly, LRRK2 kinase hyperactivation could be involved in this detrimental process, because an increased presence of the pathological phosphorylated form of α-syn, the pSer129 α-syn, was found in the striatal dopaminergic terminals of 12-month-old G2019S LRRK2 transgenic mice (Longo et al., 2017), and G2019S LRRK2 expression in cultured neurons, or in rat midbrain, was able to increase the recruitment of endogenous α-syn into pathological inclusions (Volpicelli-Daley et al., 2016). This last evidence led to the hypothesis that LRRK2 could facilitate, through its kinase activity, the progression of α-syn pathology by creating a pool of α-syn more susceptible to aggregates (Volpicelli-Daley et al., 2016), as also shown in G2019S KI mice (MacIsaac et al., 2020) and in hIPSC expressing G2019S LRRK2 (Bieri et al., 2019). In line with this, transgenic mice with a conditional expression of G2019S LRRK2 in the forebrain were characterized by kinase-dependent behavioral deficits associated with α-syn pathology in the CNS (Xiong et al., 2017), whereas cortical neurons from G2019S LRRK2 transgenic mice showed endogenous insoluble α-syn aggregates that could be reduced by the pharmacological inhibition of LRRK2 kinase activity (Schapansky et al., 2018). Moreover, a twofold higher load of pSer129 α-syn compared to wild-type animals was found in 12-month-old G2019S KI mice injected with a viral vector overexpressing human mutant A53T α-syn (Novello et al., 2018). LRRK2, as well as fragments containing its kinase domain, was hypothesized to phosphorylate recombinant α-syn at serine 129, especially in the presence of G2019S mutation (Qing et al., 2009).

However, it should be noted that subsequent studies have criticized the possible pathological interaction between LRRK2 and α-syn. Indeed, the coexpression of LRRK2 and α-syn genes was not followed by changes in the extent of the α-synucleinopathy or α-syn phosphorylation state (Herzig et al., 2012), and the overexpression of human G2019S LRRK2 did not modify the α-synucleinopathy characterizing A53T α-syn transgenic mice (Daher et al., 2012). Since the tissutal and temporal expression of LRRK2 could vary among the various experimental models analyzed, some authors have hypothesized that the LRRK2-mediated exacerbation of α-syn pathology could be cell type- and brain region-dependent (Herzig et al., 2012).

It should also be considered that LRRK2 could influence α-syn aggregation through indirect pathways. In this scenario, the Rab GTPases have been proposed as possible mediators, because they represent one of the main endogenous LRRK2 substrates and were found to be involved in LRRK2-dependent α-synucleinopathy propagation (Bae et al., 2018). In addition, the interaction between LRRK2 and Rab proteins could also influence the physiological trafficking of autophagosomes and lysosomes, which plays a key role in the removal of pathological α-syn aggregates (Dinter et al., 2016; Bellomo et al., 2020). Indeed, different authors suggested that mutant LRRK2 could impair the mechanisms leading to the clearance of pathological α-syn aggregates, such as the neuronal chaperone-mediated autophagy (Cuervo et al., 2004; Vogiatzi et al., 2008; Tong et al., 2010; Orenstein et al., 2013) or the immune-dependent clearance of α-syn aggregates (Schapansky et al., 2015). Specifically, a recent report showed that G2019S mutant LRRK2 could influence lysosomal acidification, decreasing the autophagic processes and increasing the accumulation of neuronal insoluble α-syn aggregates, which could be subsequently released in the extracellular space (Schapansky et al., 2018). Moreover, pharmacologic inhibition of LRRK2 kinase activity was able to reverse this pathological pathway (Schapansky et al., 2018).

In this scenario, of particular interest is the possibility that LRRK2 could influence the inflammatory and microglial response to progressive α-synucleinopathy within the CNS. Different studies have reported that LRRK2 acts as a regulator of microglial activation (Gillardon et al., 2012; Moehle et al., 2012; Russo et al., 2015), and the hyperactivation of its kinase domain could amplify phagocytic activity and/or proinflammatory microglial response (Kim et al., 2012, 2018; Moehle et al., 2015). An exaggerated LRRK2-dependent inflammatory response to α-syn aggregation could worsen the neuronal oxidative stress and the neurodegenerative process leading to PD (Cresto et al., 2019). Accordingly, double-transgenic G2019S/A53T mice were characterized by the presence of microgliosis and enhanced DAergic neuronal loss (Lin et al., 2009), and rats expressing G2019S LRRK2 showed an exacerbated inflammatory response to α-syn overexpression, which was reduced by LRRK2 kinase inhibition (Daher et al., 2015). Other authors have reported that microglial cells obtained from LRRK2 KO mice showed increased α-syn uptake and clearance (Maekawa et al., 2016) and that nigral or striatal microglial activation was not significantly different between transgenic G2019S LRRK2 and wild-type mice injected with a AAV–α-syn (Novello et al., 2018), suggesting the need of further investigations on the theme.

Lastly, another intriguing hypothesis that could explain the link between LRRK2 and α-syn is the possibility that mutant LRRK2 may enhance neuronal spreading of α-syn within the CNS. Indeed, cell-to-cell transmission of α-syn was investigated in G2019S LRRK2-expressing neuroblastoma cells, showing enhanced α-syn release into extracellular media (Kondo et al., 2011).

In conclusion, different studies suggested that LRRK2 and α-syn may interact in various ways during the progressive loss of striatal DAergic innervation characterizing PD. Mutant LRRK2 could influence the development of PD-related α-synucleinopathy at different time points, altering its phosphorylation, aggregation, propagation, or clearance. This could have particular relevance during the earlier phases of the disease, in which the abnormalities of striatal synaptic transmission are thought to play a crucial pathogenic role (Schirinzi et al., 2016). Indeed, it has been demonstrated that exposure to pathological α-syn oligomers is able to disrupt the expression of synaptic LTP in striatal cholinergic interneurons (Tozzi et al., 2016) and SPNs (Durante et al., 2019), through an interaction with different subunits of postsynaptic NMDAR, such as GluN2D and GluN2A, respectively. The pathological consequences of α-syn aggregates on synaptic plasticity could worsen the synaptic abnormalities uncovered in LRRK2 genetic models, leading to a diffuse disruption of striatal network functioning even before the loss of DAergic nigral cells. Despite all this, it is important to note that α-syn pathology is not present in all LRRK2 cases. Histopathological studies have shown that that a high percentage (~43%) of cases had no LB inclusions (Poulopoulos et al., 2012). Intriguingly, this seemed to be in a higher proportion in non-G2019S mutations. These human patient data make the LRRK2–α-syn interaction even more complex and warrants further study with multiple mutations.

## Conclusions and Future Perspectives

Approximately two decades ago, the characterization of the “Sagamihara family” unveiled a new unexpected and extremely intriguing field of research in neuroscience. What was initially considered a rare genetic cause of parkinsonism turned out to be a crucial PD-related pathogenic protein, potentially involved in familial and sporadic PD. Thanks to the investigation performed in genetic experimental models, the previously unknown pathophysiological functions of LRRK2 started to be understood. LRRK2 mutation seems to influence striatal synaptic transmission in an age-dependent way, through the regulation of both presynaptic vesicle release and postsynaptic receptor activity, contributing to impairment of basal ganglia network during the course of PD. The study of transgenic LRRK2 mouse models has reinforced the idea that early PD phases are characterized by diffuse, and potentially reversible, striatal synaptopathy, preceding the progressive loss of nigral cells. Indeed, the dysfunction of presynaptic DAergic terminals triggered by LRRK2 through different mechanisms, such as vesicle trafficking deregulation, mitochondrial dysfunction, and disrupted Ca^2+^ homeostasis, could be slowly followed by the degeneration of the DAergic neuronal bodies located in the SNpc. This process could be further sustained by the enhanced susceptibility to ROS and environmental stressors seen in LRRK2 mutant models. Moreover, LRRK2 is thought to favor pathological α-syn phosphorylation, aggregation, and interneuronal propagation, which could worsen itself mitochondrial activity sustaining the detrimental vicious cycle leading to nigral degeneration (Ordonez et al., 2018; Bastioli et al., 2019). Accordingly, LRRK2 could represent a molecular target for strategies counteracting the progressive α-synucleinopathy and mitochondrial impairment characterizing PD.

Efforts have been made to identify pharmacological, brain-penetrant inhibitors of LRRK2 usable as potential disease-modifying strategies. The first compounds identified as LRRK2 inhibitors were nonselective kinase inhibitors with multiple targets and potential adverse effects (Vancraenenbroeck et al., 2011; Lee B. D. et al., 2012; Taymans and Greggio, 2016; West, 2017; Chen et al., 2018). More recently, new generation LRRK2 kinase inhibitors have been developed and tested *in vitro* or *in vivo*, with improved potency, better selectivity, and/or long-term efficacy (Taymans and Greggio, 2016; West, 2017; Chen et al., 2018). It should be considered that, even if the *in vivo* or *in vitro* inhibition of LRRK2 kinase activity was able to rescue many of the detrimental neuronal effects triggered by mutant LRRK2, the systemic consequences of chronic LRRK2 inhibition should be carefully considered. For example, studies performed in LRRK2 KO models showed that the loss of LRRK2 activity could impair cellular lysosomal pathways in different organs, such as kidneys, lungs, and liver (Tong et al., 2010; Herzig et al., 2011; Baptista et al., 2013; Ness et al., 2013). The effects of LRRK2 inhibition could be tissue- and age-dependent, so the observations made in transgenic LRRK2 KO models may not reflect the complete picture of a pharmacological LRRK2 inhibition during adult age. Moreover, the phenotype of LRRK2 KO models could rely on the loss of the global protein function, beyond its kinase activity (Taymans and Greggio, 2016), reinforcing the need of a global safety/efficacy assessment of these compounds in preclinical transgenic KI models of PD. Indeed, it has been noted that not all LRRK2 PD-related mutations cause an increase in kinase activity (Rudenko et al., 2012), importantly implying that kinase function alone may not be the key (Cookson, 2015); thus, reduction of kinase in all cases could actually be detrimental.

Furthermore, the possible application in humans should be carefully planned. Indeed, many questions should be answered for an adequate evaluation of the potential benefits of these molecules in a clinical setting. First, reliable biomarkers reflecting *in vivo* LRRK2 kinase activity beyond total LRRK2 protein levels are needed. This could be crucial in identifying patients with high LRRK2 activity, independently from the genetic testing for known LRRK2 mutation, as well as to assess the efficacy of the drug and its therapeutic range in a longitudinal clinical context. In this scenario, hypothetical LRRK2 kinase substrates, such as the suggested pSer1292-LRRK2 or pRabs, could be analyzed in white blood cells and in purified exosome fractions from cerebrospinal fluid and urine to provide a surrogate measure of LRRK2 activity (West, 2017; Zhao and Dzamko, 2019). Specifically, the determination of Rab10 Thr73 phosphoepitope in neutrophils obtained from patients’ blood samples has been proposed to assess LRRK2 kinase activity *in vivo* (Thirstrup et al., 2017; Fan et al., 2018), even if with some limitations (Atashrazm et al., 2019). Moreover, other authors have proposed as LRRK2 kinase activity biomarker the analysis of centrosomal cohesion deficit in peripheral blood mononuclear cell–derived LCLs (Fernández et al., 2019), because it is dependent on phospho-Rab8 and phospho-Rab10 and can be reverted by LRRK2 inhibition (Lara Ordónez et al., 2019). Such biomarkers would allow to treat an appropriate cohort of patients, considering that not only mutant LRRK2 carriers but also a subpopulation of sporadic PD patients could benefit from LRRK2 pharmacological inhibition. Lastly, it should be defined which strategy of LRRK2 inhibitors administration could be associated with the best efficacy. Even if the administration of the drug could be started during the presymptomatic phase in mutant LRRK2 carriers, or during the early symptomatic phase in sporadic PD patients, many studies have suggested that the biological functions of LRRK2 are age-dependent and the pathological long-term changes of striatal network could be triggered very early during the postnatal life. Pharmacological LRRK2 inhibition could have minor effects if the pathogenic events leading to PD, such as progressive α-synucleinopathy or ROS-induced mitochondrial dysfunction, have been already started. To date, there is poor evidence to exclude the presence of an early, reversible, LRRK2-dependent and a late, irreversible, LRRK2-independent pathogenic phase in PD development.

Overall, the investigation of LRRK2-related PD has brought unexpected results, improving our understanding of PD pathogenesis with potential implications for a large number of patients. For now, there is the need for a differentiated research effort to reach multiple objectives and clarify this promising pathological pathway. The molecular mechanisms linking LRRK2 function, striatal synaptopathy, mitochondrial dysfunction, and progressive α-synucleinopathy should be better understood, as well as their timing and mutual relationships. The efficacy/safety ratio of LRRK2 inhibition should be clarified in transgenic models resembling human LRRK2 expression pattern and function, during both physiological and pathological conditions. Reliable biomarkers reflecting LRRK2 *in vivo* activity should be developed to identify all PD patients that could benefit from an anti-LRRK2 therapy. Solving these issues surely does not represent an easy project, but LRRK2 appears as one of the more promising targets for a neuroprotective therapy counteracting the multiple pathogenic processes underlying PD development. The chances that the observations identified in the “Sagamihara family” could turn into an effective therapy for millions of people worldwide may be small, but we should not miss this opportunity.

## Author Contributions

AMa, AT, and PC conceived the review. AMa, AT, and DB-K performed literature review. AMa wrote the manuscript draft and prepared the figures. AT, DB-K, MDF, and PC reviewed the manuscript draft and the figures. All authors read and approved the final manuscript.

## Conflict of Interest

AMa received travel grants from Biogen, Biogen-Idec, Novartis, Merck, Teva, Almirall and Sanofi Genzyme to attend national and international conferences. MDF participated on advisory boards for and received speaker or writing honoraria and funding for travelling from Bayer, Biogen Idec, Genzyme, Merck, Novartis, Roche and Teva. PC participated on advisory boards for and received funding for travelling, speaker honoraria and research support from AbbVie, Biogen Idec, Merck, Genzyme, Novartis, Prexton, Teva, UCB and Zambon. The remaining authors declare that the research was conducted in the absence of any commercial or financial relationships that could be construed as a potential conflict of interest.
